# Clinical characteristics of survivors versus non-survivors after acute diquat poisoning: a comparative study

**DOI:** 10.1007/s11739-023-03460-z

**Published:** 2023-12-08

**Authors:** Min Yan, Hongbo Liu, Yihong Yang, Xin Cheng, Wanpeng Sun, Tengfei Ma, Xiaopei Cai

**Affiliations:** Emergency Department, Fuyang People’s Hospital, Fuyang, 236000 China

**Keywords:** Diqaut poisoning, Prognosis, Survive, Death, Comparative study

## Abstract

The aim of this study was to compare the clinical characteristics between survivors and non-survivors after acute diquat (DQ) poisoning. Patients treated in the Emergency Department of Fu Yang People’s Hospital for DQ poisoning between January 2018 and February 2022 were enrolled in this retrospective comparative study. A total of 65 patients were collected, including 36 males (55.4%) and 29 females (44.6%). There were 34 survivors (52.3%), and 31 non-survivors (47.7%). Patients in the non-survivor group were significantly older (*P* = 0.003), received a higher dose of DQ before admission (*P* < 0.001), had more severe organ damage (*P* < 0.001), lower respiration rate (*P* < 0.001) and enema (*P* = 0.009), lower GCS score (*P* = 0.038), and higher SIRS score (*P* = 0.018) and APACHE-II score (*P* < 0.001) than patients in the survivor group. Additionally, biochemical indicators after admission between survivors and non-survivors were significantly different (all *P *< 0.05). Multivariate logistic regression analysis showed that respiratory failure (*P* = 0.021), the dose of DQ (*P* = 0.022), respiratory rate (*P* = 0.007), and highest alanine transaminase (ALT) level after admission (*P* = 0.030) were independent risk factors for acute DQ-induced death. These data suggest that non-survivors with acute DQ poisoning are more likely to suffer from respiratory failure, have higher respiratory rate and ALT after admission, and are exposed higher doses of DQ before admission than survivors.

## Introduction

Diquat (DQ) (6,7-dihydrodipyrido [1,2-a: 2, 1’-c] pyrazine-5, 8-diium dibromide) and paraquat are rapidly acting contact herbicides. The marketing of DQ started in 1958, earlier than that of paraquat; yet, compared to paraquat, the market promotion of DQ was slower and its utilization rate was lower. Interestingly, between 1998 and 2013, the United States (US) toxicology database reported 693 patients hospitalized for paraquat poisoning and 2128 patients hospitalized due to DQ poisoning in the USA [1], which may due to the restrictions on the production and sale of paraquat in most countries; for example, European Union (EU) [2], China [3] and at least 7 other countries [4] have banned the sale and use of paraquat. Consequently, the use of DQ has been gradually increasing in these countries [1, 5], which led to a higher rate of DQ poisoning.

In 2005, the World Health Organization (WHO) reported that DQ is a pesticide with moderate toxicity [6], and its toxicity is lower than paraquat (which is regarded as a highly toxic pesticide). Poisoning from DQ mainly occurs from accidental ingestion, and most clinical manifestations result from oxidative stress due to the accumulation of reactive oxygen species [7]. The major clinical manifestations of DQ poisoning include erosion of mucus membranes, acute renal failure, cardiovascular and cerebral vascular complications (brainstem infarction, cerebral hemorrhage, and circulatory failure), and multiple organ failure (MOF), which may be lethal [1, 8].

Thus far, no specific antidote has been found for DQ poisoning. Due to multiple organ involvement, patients can present with various manifestations, further challenging the treatment [9]. Also, as patients show variation in elevation of biochemical indices, there are currently no biomarkers for assessing the disease severity or prognosis [10]. However, several studies have shown that early intervention and quick halting of the compound absorption into the body can prevent MOF [11, 12]. Furthermore, some authors have successfully treated DQ poisoning using traditional Chinese medicine [13]. Moreover, no universally acknowledged standard criteria have been developed to evaluate the clinical characteristics of acute DQ poisoning and predict mortality.

This study aimed to compare the clinical characteristics between survivors and non-survivors after acute diquat (DQ) poisoning.

## Methods

### Study design and population

This comparative study enrolled patients with DQ poisoning treated in the Emergency Department of Fu Yang People’s Hospital between January 2018 and February 2022. The inclusion criteria were as follows: (1) patients with DQ poisoning; (2) complete clinical data including patient's clinical characteristics and biochemical indicators. The exclusion criteria were: (1) history of systemic rheumatic diseases; (2) previous history of one or more chronic organ dysfunction; (3) immune disorders.

This study was approved by the Ethics Committee of Fu Yang People’s Hospital. The committee waived the requirement for informed consent due to the nature of the retrospective study.

### Data collection and definition

The following clinical characteristics of the patients were collected, including gender, age, occupation (Table 1), underlying disease, alcohol consumption; time interval from poisoning to hospital admission, type and dose of poisoning, vital signs on admission, gastric lavage, catharsis, enema, blood transfusion, time from poisoning to treatment; the renal, hepatic, myocardial and pulmonary function, coagulation function; Glasgow Coma Scale (GCS), systemic inflammatory response syndrome (SIRS) score, acute physiology and chronic health evaluation-II (APACHE-II) score. Biochemical indicators [white blood cell (WBC), hematocrit (HCT), prothrombin time (PT), thrombin time (TT), D-dimer (D-D), glucose (GLU), lactate dehydrogenase (LDH), creatine kinase (CK), kinase-MB isoform (CK-MB), kalium (K), sodium (NA), CO2/(HCO3), blood urea nitrogen (BUN), creatinine (CREA), and C-reactive protein (CRP)] were collected immediately at the time of admission, the highest value (WBC, PT, TT, D-D, GLU, LDH, CK, CK-MB, K, NA, CO2/(HCO3), BUN, CREA, and CRP) and lowest value (HCT, PT, TT, GLU, K, NA, and CO2/(HCO3)) were collected after admission. Time at admission refers to the value of the first measurement within 2 h after admission; lowest value after admission refers to the lowest measurement during the treatment processes after admission; highest value after admission refers to the highest measurement during the treatment processes.

**Table 1:** Occupational characteristics of 64 patients with acute Diquat poisoning

Occupations	Survival group (*n* = 34) %	Non-survival group (*n* = 31)
Farmer	20 (58.82)	19 (61.29)
Student	7 (20.59)	1 (3.22)
Worker	1 (2.94)	3 (9.68)
Others	6 (17.65)	8 (25.81)

Acute DQ poisoning refers to cases with one overdose or high dose exposure of DQ that induced diseases or death due to structural and functional damages to tissues or metabolic disorders. Respiratory failure refers to the severe pulmonary ventilation and/or respiratory dysfunctions caused by various causes, leading to the incapability of effective gas exchange, hypoxia accompanied with (or without) CO_2_ retention, and consequently causing acute respiratory distress clinical syndrome and metabolic disturbance. Organ damage refers to visceral function abnormalities, such as renal, hepatic, pulmonary or cardiac dysfunction. The time interval from poisoning to treatment refers to the time from oral intake of DQ to being treated in the Emergency Department of Fu Yang People’s Hospital.

GCS score can be used to judge and assess the degree of coma in the patients and help make an appropriate diagnosis and treatment [14]. The GCS includes three aspects: eye-opening, verbal, and motor response. The summary of scores of the 3 aspects is the coma index, an indicator for evaluating the degree of coma in patients in clinical practices. SIRS score reflects the pathophysiological status induced by the over-secretion of various inflammatory mediators and the over-activation of inflammatory cells after exposure to infectious or non-infectious agents [15]. The SIRS score is used to assess the disease condition in the post-operative care of critical patients who underwent surgery. The APACHE-II is a severity-of-disease scoring system [16] that consists of 3 parts: acute physiology, age, and chronic health conditions. The total score of APACHE-II is calculated by adding the scores from the 3 parts. MOF is defined as the failure of ≥ 3 organs.

Patients were categorized into survivor and non-survivor groups according to the final outcomes. Patients who died during hospitalization or within 2 months after discharge were classified as the non-survivor group. Death was diagnosed according to cardiac and respiratory arrest markers and brain death.

### Statistical analysis

IBM SPSS Statistics, version 22.0 (IBM Corp., Armonk, N.Y., USA) was used for statistical analyses. Continuous data with normal distribution and equal variances were described by mean and standard deviation (SD). An independent t-test was used for group comparison. Continuous data with skewed distribution were described by median (interquartile range: P25, P75), and group comparison was performed with a non-parametric rank-sum test. Categorical data were described by percentages (%) and compared using the chi-square test. Multivariate logistic regression analysis was performed to explore the independent risk factors of the death. A two-sided P < 0.05 was considered statistically significant.

## Results

A total of 65 patients with acute diquat poisoning were included, including 35 males (58.8%) and 29 females (44.6%). There were 34 survivors (52.30%) and 31 non-survivors (47.70%). Patients in the non-survivor group were significantly older than patients in the survivor group (39.90 ± 15.40 vs. 28.65 ± 13.48, *P* = 0.003). The percentage of patients with ≥ 5 damaged organs was significantly higher in the non-survivor group than that in the survivor group [19 (61.29%) vs. 2 (5.88%), *P* < 0.001). Respiration rate (17.62 ± 1.23 vs. 20.13 ± 3.51, *P* < 0.001) and enema [23 (67.60%) vs. 29 (93.50%), *P* = 0.009] of patients in the non-survivor group were significantly lower than that in the survivor group. Compared with the non-survivor group, the GCS score was significantly higher, and the SIRS score and APACHE-II score were significantly lower in the survivor group (all *P* < 0.05)** (**Table 2**).**

**Table 2:** Comparison of the clinical characteristics of patients with DQ poisoning between the survival and death groups

Characteristic	Survival group (*n* = 34)	Death group (*n* = 31)	*P*-value
Age (year)	28.65 ± 13.48	39.90 ± 15.40	0.003
Male (*n)*	20 (58.80%)	15 (48.40%)	0.399
Underlying disease (*n*)	8 (23.53%)	12 (38.71%)	0.185
Number of damaged organs ≥ 5 (*n*)	2 (5.88%)	19 (61.29%)	< 0.001
Alcohol drinking (*n*)	10 (29.40%)	8 (25.80%)	0.746
HR (bpm)	86.62 ± 14.81	98.90 ± 27.49	0.027
Respiration rate (bpm)	17.62 ± 1.23	20.13 ± 3.51	< 0.001
MAP (mmHg)	99.42 ± 15.61	99.88 ± 21.32	0.921
Gastric lavage	29 (85.30%)	30 (96.80%)	0.243
Catharsis	18 (52.90%)	23 (74.20%)	0.076
Enema	23 (67.60%)	29 (93.50%)	0.009
Blood perfusion	26 (76.50%)	29 (93.50%)	0.118
DQ dose (mL)	50.00 (20.00, 65.00)	137.30 (100.00, 200.00)	< 0.001
The time interval from poisoning to hospital admission (h)	7.00 (4.75, 24.00)	5.00 (4.00, 8.00)	0.049
Hospital stay (days)	6.00 (1.75, 12.75)	2.00 (1.00, 7.00)	0.057
GCS score	14.85 ± 0.61	13.74 ± 3.00	0.038
SIRS score	2.00 (1.00, 4.00)	4.00 (2.00, 6.00)	0.018
APACHE-II score	4.00 (3.00, 7.00)	7.00 (6.00, 11.00)	< 0.001

*HR* heart rate, *MAP* mean arterial pressure, *DQ* Diquat, *GCS* Glasgow Coma Scale, *SIRS* systemic inflammatory response syndrome, *APACHE-II* acute physiology and chronic health evaluation-II

Additionally, the comparison of the biochemical indicators including ALT, AST, LDH, CK, CK-MB, BUN, CRP, WBC and highest WBC count after admission; LDH, CK, CK-MB, BUN, CRP, and CO_2_/(HCO_3_^−^) and lowest CO_2_/(HCO_3_^−^) level after admission were different between survivor and non-survivor patients (all *P* < 0.05)** (**Table 3**).**

**Table 3:** Comparison of various laboratory indicators after admission between the survival and death groups

Biochemical indicator after admission	Survival group (*n* = 34)	Death group (*n* = 31)	*P* value
Immediate WBC (*10^9^/L)	12.01 ± 5.70	15.48 ± 7.49	0.039
Highest WBC (*10^9^/L)	18.35 ± 8.24	22.95 ± 8.34	0.029
Immediate HCT (%)	42.33 ± 7.10	42.95 ± 4.89	0.689
Lowest HCT (%)	32.13 ± 7.81	35.55 ± 8.36	0.093
Immediate PT (*10^9^/L)	13.00 (12.30, 13.73)	13.30 (12.20, 13.90)	0.442
Highest PT (*10^9^/L)	13.95 (12.78, 16.48)	15.40 (13.50, 20.50)	0.035
Lowest PT (*10^9^/L)	12.39 ± 2.22	13.05 ± 1.97	0.218
Immediate TT(S)	18.51 ± 1.88	18.63 ± 2.68	0.838
Highest TT (S)	21.55 (18.50, 162.50)	20.60 (19.00, 170.00)	0.833
Lowest TT (S)	17.71 ± 1.96	17.97 ± 1.70	0.573
Immediate D-D (mg/L)	0.30 (0.20, 0.41)	0.34 (0.20, 0.90)	0.366
Highest D-D (mg/L)	0.76 (0.37, 1.82)	0.81 (0.34, 2.00)	0.698
Immediate GLU (mmol/L)	6.52 ± 1.74	7.53 ± 3.67	0.158
Highest GLU (mmol/L)	10.20 ± 3.19	10.81 ± 5.70	0.590
Lowest GLU (mmol/L)	5.21 ± 1.40	6.23 ± 2.73	0.060
Immediate LDH (U/L)	387.50 (241.03, 523.90)	555.70 (352.00, 750.30)	0.023
Highest LDH (U/L)	473.80 (255.88, 859.30)	1424.10 (560.20, 2457.00)	< 0.001
Immediate CK (U/L)	107.35 (68.30, 144.88)	156.00 (83.80, 492.90)	0.032
Highest CK (U/L)	129.90 (99.68, 452.50)	588.00 (206.00, 4606.00)	0.002
Immediate CK-MB (ng/ML)	17.45 (10.38, 26.13)	28.00 (19.70, 47.00)	0.005
Highest CK-MB (ng/ML)	22.85 (18.58, 43.95)	60.30 (27.00, 254.20)	< 0.001
Immediate K (mmol/L)	3.77 ± 0.43	3.63 ± 0.75	0.355
Highest K (mmol/L)	4.47 ± 0.58	4.79 ± 1.19	0.176
Lowest K (mmol/L)	3.13 ± 0.48	3.22 ± 0.69	0.531
Immediate NA (mmol/L)	137.79 ± 3.23	137.35 ± 4.18	0.635
Highest NA (mmol/L)	141.33 ± 5.07	142.95 ± 9.21	0.375
Lowest NA (mmol/L)	132.47 ± 5.38	126.88 ± 24.79	0.204
Immediate CO_2_/(HCO_3_) (mmol/L)	23.22 ± 4.66	18.82 ± 5.35	0.001
Highest CO_2_/(HCO_3_) (mmol/L)	28.37 ± 3.91	22.64 ± 7.59	< 0.001
Lowest CO_2_/(HCO_3_) (mmol/L)	18.55 ± 4.61	14.17 ± 5.10	0.001
Immediate BUN (mmol/L)	4.90 (3.28, 6.70)	6.30 (4.40, 9.20)	0.048
Highest BUN (mmol/L)	11.35 (5.30, 23.43)	13.40 (9.10, 26.40)	0.088
Immediate CREA (μmol/L)	64.70 (51.98, 106.38)	71.20 (59.20, 169.20)	0.235
Highest CREA (μmol/L)	102.30 (58.35, 352.25)	296.20 (205.00, 504.10)	0.002
Immediate CRP (mg/L)	5.00 (3.05, 6.92)	5.50 (5.00, 23.26)	0.021
Highest CRP (mg/L)	9.68 (2.71, 27.25)	31.30 (8.33, 88.20)	0.020

Immediate after admission refers to the value of the first measurement within 2 h after admission; lowest after admission refers to the lowest measurement during the treatment processes after admission; highest after admission refers to the highest measurement during the treatment processes after admission. *WBC* white blood cell; *HCT* red blood cell-specific volume, *PT* prothrombin time, *TT* thrombin time, *D-D* D-Dimer, *GLU* glucose, *LDH* lactate dehydrogenase, *CK* creatine kinase, *CK-MB* creatine kinase-MB isoform, *BUN* blood urea nitrogen, *CREA* creatinine, *CRP* C-reactive protein

Furthermore, multivariate logistic regression analysis showed that respiratory failure (OR = 171.002; 95% CI 2.186–13,379.829; *P* = 0.021), the dose of DQ (OR = 1.038; 95% CI 1.005–1.071; *P *= 0.022), respiratory rate (OR = 15.089; 95% CI 2.106–108.117; *P* = 0.007), and highest alanine transaminase (ALT) level after admission (OR = 1.004; 95% CI 1.000–1.007; *P *= 0.030) were independent risk factors for acute DQ-induced death **(**Table 4**)**.

**Table 4:** Multivariate logistic regression analysis screening potential lethal factors for patients with DQ poisoning

Variables	OR (95%CI)	*P* value
Respiratory failure (*N*)	171.002 (2.186–13,379.829)	0.021
DQ dose (ML)	1.038 (1.005–1.071)	0.022
Respiration rate (bpm)	15.089 (2.106–108.117)	0.007
Highest ALT after admission (U/L)	1.004 (1.000–1.007)	0.030

## Discussion

The present study showed that respiratory failure, higher respiratory rate, and higher ALT after admission, as well as taking higher doses of DQ before admission, are critical risk factors that may be lethal to patients with acute DQ poisoning. This study provides a clue on assessing the severity of DQ poisoning and obtaining markers for monitoring treatment response and prognostication.

Renal failure is the most common manifestation in patients with DQ poisoning [17, 18], which was also observed in patients in the present study. Despite sufficient fluid supplementation and achieving normal arterial and central venous pressure, renal failure was observed, suggesting renal toxicity induced by DQ. Although there is no specific antidote for DQ or paraquat poisoning, treatments are generally similar and entail prompt absorption prevention and enhancement of the elimination of the poison [19]. Gastric lavage with an oral gastric tube, preferably with water or soap water or 1% to 2% sodium bicarbonate solution, is recommended. Gastric lavage should be as thorough as possible, with a general gastric lavage solution of ≥ 5 L until colorless and odorless. Immediately after gastric lavage, an injection of 15% bleach solution of adsorbent (1000 mL of adult total) or activated carbon (50–100 g of adult) is recommended. Catheterization should be performed as follows: 20% mannitol, sodium sulfate, or magnesium sulfate can be given to promote the excretion of intestinal toxins and reduce absorption. Afterward, patients can continuously take bleached soil or activated carbon orally for 2–3 days to induce diarrhea. Water enema should be cleared every 4–6 h for 2–3 consecutive days. The absorption of residual DQ in the gastrointestinal tract could lead to the accumulation of DQ in the circulation, organs, and tissues, leading to multiorgan damage and increasing the mortality risk [20]. Thus, prompt gastrointestinal decontamination can reduce MOF risk [21]. The treatment is given to counteract the mechanisms of action of DQ (and paraquat), which is mainly caused by oxidative stress [22]. When DQ is absorbed into circulation, large amounts of superoxide anion free radicals are generated through oxidative stress and lipid peroxidation, damaging cells and tissues [23].

Previous studies have shown that death from DQ poisoning is mainly caused by MOF [24]. Almost all the patients who died from DQ poisoning in this study had MOF. The CNS manifestations in our patients included agitation, delirium, and lethargy, which have been previously reported [25]. Imaging examinations of patients who died shortly after DQ poisoning demonstrated the presence of severe CNS damage [12, 18] from acute toxic encephalopathy and cerebral hemorrhage. Additionally, oxidative stress on the heart may lead to severe myocardial injury, ischemia, arrhythmia, significant elevation of myocardial enzymes, refractory cardiogenic shock, and sudden death in the patients. The myocardium requires intense energy metabolism and thus could be one of the most sensitive target organs for DQ-induced oxidative damage [26]. Nonetheless, the clinical manifestations and outcome of DQ poisoning differ from those of paraquat poisoning, as DQ has a fivefold shorter half-life than paraquat and rarely causes long-term sequelae, such as pulmonary fibrosis [22].

With the prevalence of acute DQ poisoning rising yearly, especially among farmers [1], improving rural education about the dangers of DQ exposure is critical. In clinical practice, it is challenging for doctors in emergency departments to evaluate the DQ dose according to the information in medical records. Some patients may be unable to clearly describe the DQ dose, while some may even deny the DQ intake. Most patients tend to vomit after oral intake, and thus, the DQ poisoning dose may not be precisely estimated. Our data showed that patients who died from the poisoning had an elevation in WBC, PT, LDH, CK, CK-MB, CRP and CREA, and lower CO_2_/(HCO_3_) during management. Elevation of these biomarkers reflects multiple organ damage and may be helpful when categorizing the severity of poisoning and monitoring treatment responses.

The present study has several limitations. Currently, the liquid preparations of DQ in the market are mixed with different concentrations of paraquat [27, 28], which could lead to unexpected clinical manifestations in patients and substantially influence the clinical diagnosis and treatment of DQ poisoning. As a result, our department obtained a high-performance liquid chromatography mass spectrometer in January 2020 to rule out patients with paraquat poisoning or mixed poisoning. Thus, our study included only DQ poisoning patients, allowing for more accurate clinical diagnosis and outcome prediction. This was a single-center study with a small sample size, so a multi-center study with a larger sample size is needed.

In summary, non-survivors with acute DQ poisoning were more likely to suffer from respiratory failure, have higher respiratory rate and higher ALT after admission, and have taken higher doses of DQ before admission than survivors.

## Data Availability

The data supporting the findings of this study are available on request from the corresponding author.
